# Circulating Tumor DNA Profiling in Liver Transplant for Hepatocellular Carcinoma, Cholangiocarcinoma, and Colorectal Liver Metastases: A Programmatic Proof of Concept

**DOI:** 10.3390/cancers16050927

**Published:** 2024-02-25

**Authors:** Hanna Hong, Chase J. Wehrle, Mingyi Zhang, Sami Fares, Henry Stitzel, David Garib, Bassam Estfan, Suneel Kamath, Smitha Krishnamurthi, Wen Wee Ma, Teodora Kuzmanovic, Elizabeth Azzato, Emrullah Yilmaz, Jamak Modaresi Esfeh, Maureen Whitsett Linganna, Mazhar Khalil, Alejandro Pita, Andrea Schlegel, Jaekeun Kim, R. Matthew Walsh, Charles Miller, Koji Hashimoto, David Choon Hyuck Kwon, Federico Aucejo

**Affiliations:** 1 Department of Hepato-Pancreato-Biliary & Liver Transplant Surgery, Digestive Diseases and Surgery Institute, Cleveland Clinic Foundation, Cleveland, OH 44195, USAzhangm5@ccf.org (M.Z.); dmg173@case.edu (D.G.); khalilm5@ccf.org (M.K.); schlega4@ccf.org (A.S.);; 2 Department of Hematology and Oncology, Taussig Cancer Institute, Cleveland Clinic Foundation, Cleveland, OH 44195, USA; 3 Molecular Pathology and Cytogenomics, Pathology and Laboratory Medicine Institute, Cleveland Clinic Foundation, Cleveland, OH 44195, USA; azzatoe@ccf.org; 4 Department of Gastroenterology, Hepatology, and Nutrition, Digestive Diseases and Surgery Institute, Cleveland Clinic Foundation, Cleveland, OH 44195, USAlinganm@ccf.org (M.W.L.)

**Keywords:** liver transplant, circulating tumor DNA, liquid biopsy, liver cancer, hepatocellular carcinoma, cholangiocarcinoma, colorectal liver metastasis

## Abstract

**Simple Summary:**

Circulating tumor DNA (ctDNA) is emerging as a diagnostic and surveillance tool in cancer and recurrence. The recurrence rates after liver transplant for cancer are significant, highlighting the need for early detection and treatment. We report a cohort of patients who underwent liver transplant for hepatocellular carcinoma, cholangiocarcinoma, or colorectal cancer liver metastasis and received ctDNA testing pre- and/or post-transplant. We aim to show how ctDNA testing can be incorporated into pre-transplant work-up and post-transplant surveillance and discuss the benefits of this testing modality in the identification of genetic targets and surveillance of recurrence.

**Abstract:**

Introduction: Circulating tumor DNA (ctDNA) is emerging as a promising, non-invasive diagnostic and surveillance biomarker in solid organ malignancy. However, its utility before and after liver transplant (LT) for patients with primary and secondary liver cancers is still underexplored. Methods: Patients undergoing LT for hepatocellular carcinoma (HCC), cholangiocarcinoma (CCA), and colorectal liver metastases (CRLM) with ctDNA testing were included. CtDNA testing was conducted pre-transplant, post-transplant, or both (sequential) from 11/2019 to 09/2023 using Guardant360, Guardant Reveal, and Guardant360 CDx. Results: 21 patients with HCC (*n* = 9, 43%), CRLM (*n* = 8, 38%), CCA (*n* = 3, 14%), and mixed HCC/CCA (*n* = 1, 5%) were included in the study. The median follow-up time was 15 months (range: 1–124). The median time from pre-operative testing to surgery was 3 months (IQR: 1–4; range: 0–5), and from surgery to post-operative testing, it was 9 months (IQR: 2–22; range: 0.4–112). A total of 13 (62%) patients had pre-transplant testing, with 8 (62%) having ctDNA detected (ctDNA+) and 5 (32%) not having ctDNA detected (ctDNA-). A total of 18 (86%) patients had post-transplant testing, 11 (61%) of whom were ctDNA+ and 7 (33%) of whom were ctDNA-. The absolute recurrence rates were 50% (*n* = 5) in those who were ctDNA+ vs. 25% (*n* = 1) in those who were ctDNA- in the post-transplant setting, though this difference was not statistically significant (*p* = 0.367). Six (29%) patients (HCC = 3, CCA = 1, CRLM = 2) experienced recurrence with a median recurrence-free survival of 14 (IQR: 6–40) months. Four of these patients had positive post-transplant ctDNA collected following diagnosis of recurrence, while one patient had positive post-transplant ctDNA collected preceding recurrence. A total of 10 (48%) patients had sequential ctDNA testing, of whom *n* = 5 (50%) achieved ctDNA clearance (+/−). The remainder were ctDNA+/+ (*n* = 3, 30%), ctDNA−/− (*n* = 1, 10%), and ctDNA−/+ (*n* = 1, 11%). Three (30%) patients showed the acquisition of new genomic alterations following transplant, all without recurrence. Overall, the median tumor mutation burden (TMB) decreased from 1.23 mut/Mb pre-transplant to 0.00 mut/Mb post-transplant. Conclusions: Patients with ctDNA positivity experienced recurrence at a higher rate than the ctDNA- patients, indicating the potential role of ctDNA in predicting recurrence after curative-intent transplant. Based on sequential testing, LT has the potential to clear ctDNA, demonstrating the capability of LT in the treatment of systemic disease. Transplant providers should be aware of the potential of donor-derived cell-free DNA and improved approaches are necessary to address such concerns.

## 1. Introduction

Liver transplant is the only curative-intent treatment option for patients with unresectable hepatocellular carcinoma, cholangiocarcinoma, and colorectal liver metastasis [1,2,3,4,5,6]. However, the recurrence rates may be as high as 20–50% in certain conditions, highlighting the need for thorough and frequent post-transplant monitoring [3,4,7,8,9]. Traditional surveillance relies on cross-sectional imaging and serum biomarkers such as alpha-fetoprotein (AFP), carbohydrate/cancer antigen 19-9 (CA19-9), or carcinoembryonic antigen (CEA) depending on the disease. However, post-transplant recurrence remains a challenge in terms of diagnostics and treatment due to the limited sensitivity and specificity of current tools for cancer detection [10,11,12,13,14].

To address this issue, ctDNA-based liquid biopsy has emerged as a non-invasive approach that allows for the real-time monitoring of tumor dynamics, detection of minimal residual disease, and identification of actionable mutations [15,16,17,18,19]. In patients undergoing liver transplant, the use of cell-free DNA has also been applied to detecting rejection [20]. We have previously reported the use of ctDNA in patients undergoing liver transplant for CRLM [21]. However, its use as a predictive tool of recurrence in liver transplant remains to be fully explored.

Herein, we present a cohort of patients who underwent liver transplant for HCC, CCA, or CRLM and ctDNA testing at pre-transplant and/or post-transplant time points, demonstrating a proof of concept for ctDNA in this setting.

## 2. Methods

Patients who underwent liver transplantation for CRLM, HCC, or CCA with pre-transplant and/or post-transplant ctDNA assessment between November 2019 and September 2023 at a single quaternary care academic institution were included in this study. All the patients were evaluated by a multidisciplinary liver tumor board and liver transplant review committee. The demographic and clinical variables, including on imaging, laboratory values, and treatment courses, were collected via a retrospective review of the patients’ health charts, as approved by the Institutional Review Board (IRB).

The ctDNA was assessed using Guardant360, Guardant360 CDx, and Guardant Reveal assays (Guardant Health, Redwood City, CA, USA). Guardant360 uses next-generation sequencing (NGS) to detect clinically relevant genomic alterations in the circulating tumor DNA in plasma collected via the peripheral blood. NGS testing was performed as part of the standard clinical care in a CLIA-certified and College of American Pathologists-accredited laboratory. The blood was collected in two to four 10 mL Streck tubes, and the processed plasma was evaluated for single-nucleotide variants (SNVs), insertions–deletions (indels), gene fusions/rearrangements, and copy number variants (CNVs) across 83 genes. Mutations were annotated using OncoKB to define pathogenic variants. The blood tumor mutational burden (bTMB) was determined by analyzing the somatic SNVs and indels across a 1.0 Mb genomic backbone. For the TMB algorithm, common cancer drivers and resistance alterations, as well as putative CHIP alterations, were filtered from the analysis. Guardant Reveal uses NGS to determine the presence of ctDNA by assessing somatic alterations (SNVs, insertion–deletion alterations) and epigenomic signatures (methylation status). Guardant Reveal was used for the portion of patients with CRLM, while Guardant360 CDx was used for the portion of patients with HCC. Guardant360 was used for all cancer types.

**Figure 1:**
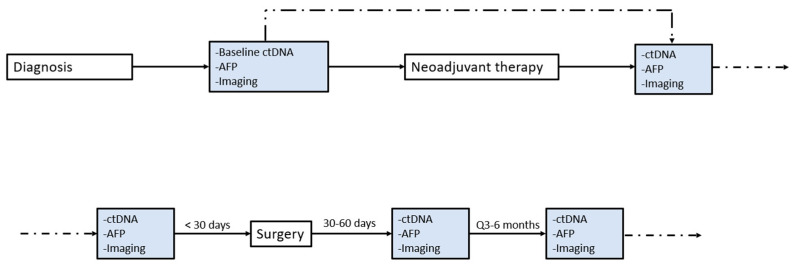
Timeline for ctDNA testing, cancer work-up, and surveillance as per institutional protocol. Note the example tumor marker shown is AFP for HCC; for other cancer types, the corresponding serum tumor marker (CCA: CA19-9, CRLM: CA19-9) is used for assessment.

Prior to January 2021, the ctDNA was collected and evaluated at the discretion of the treating surgeon. From January 2021 onward, attempts were made to collect the ctDNA at times outlined by the current institutional protocol of within 30 days pre-operatively, 30–60 days post-operatively, and every 3–6 months afterward (Figure 1). Synonymous mutations were excluded from the analysis.

Discrete variables were presented as frequency and percentages, and continuous variables were presented as medians with interquartile ranges due to non-normal distributions. Statistical analysis was performed using IBM SPSS Statistics Version 29.0 (Armonk, New York, NY, USA). A two-sided *p*-value < 0.05 was considered significant for all tests.

## 3. Results

A total of 21 patients underwent ctDNA testing and LT for HCC (*n* = 9, 43%), CRLM (*n* = 8, 38%), CCA (*n* = 3, 14%), and mixed HCC/CCA (*n* = 1, 5%) (Table 1). Nine (43%) patients underwent living donor liver transplant (LDLT), seven (33%) underwent orthotopic liver transplant (OLT) with grafts from donation after brain death (DBD), and five (24%) had OLT with grafts from donation after cardiac death (DCD) (Table 1). Most patients had cirrhosis (*n* = 19, 90%), with a median MELD score of 15 at the time of transplant. NASH (*n* = 6, 30%) was the most frequent cause of cirrhosis. The median tumor marker levels at the time of liver cancer diagnosis were AFP = 8 ng/mL (HCC), CA19-9 = 23 U/mL (CCA), and CEA = 31 ng/mL (CRLM). (Table 1). Prior to transplant, most patients (*n* = 18, 86%) received treatment, the most common being chemotherapy (*n* = 10, 48%), radiation (*n* = 6, 29%), TACE (*n* = 6, 29%), and TARE (*n* = 5, 24%). The post-transplant tumor marker levels were 3 ng/mL, 13 U/mL, and 1.8 ng/mL for AFP, CA19-9, and CEA, respectively (Table 1). Six (29%) patients (HCC = 3, CCA = 1, CRLM = 2) experienced recurrence with a median recurrence-free survival of 14 (IQR: 6-40) months. Two (10%) patients experienced cancer-related death, both with a diagnosis of HCC (Table 1). Overall, four (19%) patients experienced mortality, with a median overall survival of 16 (IQR: 8–40) months. The median and maximum follow-up times were 15 and 124 months, respectively (Table 1).

In terms of transplant, most patients (*n* = 18, 86%) underwent the piggyback technique (Table 2). The median warm ischemia time was 43 (IQR: 39–46) minutes, with a median LT duration of 589 (IQR: 471–702) minutes. Post-operatively, most patients underwent induction immunosuppression with basiliximab (*n* = 12, 57%) and initial immunosuppression with glucocorticoids, mycophenolate mofetil, and tacrolimus (*n* = 19, 90%) (Table 3). Five (24%) patients experienced bile leaks, requiring ERCP and/or PTHC, while three (14%) patients experienced biliary strictures requiring ERCP (Table 3). One patient had ischemic cholangiopathy and hepatic artery stenosis in addition to their biliary leak, requiring HJ reconstruction, PTHC, re-transplant, and stent placement (Table 3). Two patients experienced mild acute rejection, which was treated with IV steroids. No patients experienced chronic rejection (Table 3).

A total of 18 (86%) patients had post-transplant ctDNA, with 11 having ctDNA detected and 7 not having ctDNA detected (Table 4). The absolute recurrence rates were higher in patients with detected ctDNA (*n* = 5, 50%) compared to patients without ctDNA detected (*n* = 1, 25%), although this difference was not found to be statistically significant (*p* = 0.367).

Of the six (29%) patients with recurrence, five patients had post-transplant ctDNA detected. The remaining patient (#20) did not have post-transplant ctDNA detected during or after treatment of their recurrence with metastasectomy and chemotherapy (Table 4 and Table 5). Of the post-transplant ctDNA+ patients, 4/5 had ctDNA detected following radiologic detection of recurrence, while 1/5 (#7) had ctDNA detected prior to recurrence, without elevated tumor markers. Overall, only 3/6 patients (#3, 12, 21) had elevated serum tumor markers preceding recurrence, while 3/6 (#6, 7, 20) patients lacked elevation of the traditionally used tumor markers prior to recurrence.

Of all 21 patients, 10 (48%) patients had sequential ctDNA testing, with half (*n* = 5, 50%) having ctDNA clearance (+/−). The remainder were ctDNA+/+ (*n* = 3, 30%), ctDNA−/− (*n* = 1, 10%), and ctDNA−/+ (*n* = 1, 10%). More specifically, patients #9, 11, 15, 16, and 18 were ctDNA+/−; patients #2, 17, and 21 were ctDNA +/+; patient #13 was ctDNA−/−; and patient #14 was ctDNA−/+. Of note, patient #21 experienced recurrence. Three (30%) patients showed the acquisition of new genomic alterations in post-transplant ctDNA (#2, 14, 17) (Table 6). Patients #14 and 17 had no evidence of histopathologic viable tumors on explant (Table 7), suggesting potential alternate sources of these ctDNA findings. No signs of acute rejection were noticed for these patients (#14, #17, Table 6). 

**Figure 2:**
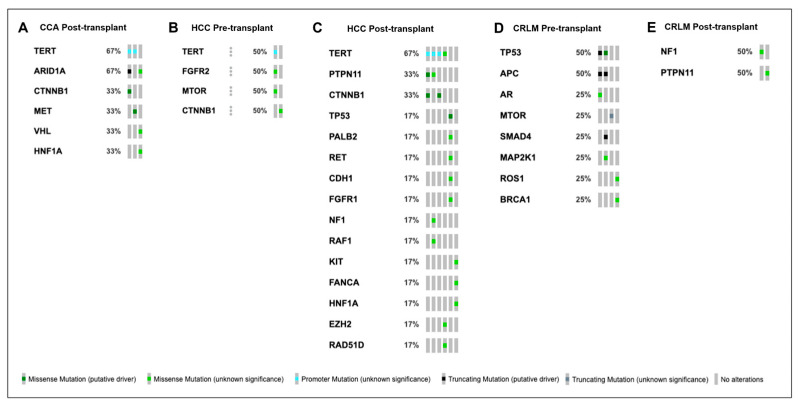
Oncoprints of genomic alterations detected using Guardant 360 available for CCA post-transplant (**A**), HCC pre-transplant (**B**), HCC post-transplant (**C**), CRLM pre-transplant (**D**), and CRLM post-transplant (**E**). Type of genomic alteration represented by color with key at bottom of figure.

Overall, the median TMB decreased from 1.23 mut/Mb pre-transplant (*n* = 9) to 0.00 mut/Mb post-transplant (*n* = 11). For HCC, a *TERT* promoter mutation was the most common genomic alteration both pre-transplant and post-transplant (Figure 2). For CRLM, *TP53* and *APC* mutations were the most common alterations observed pre-transplant, compared to *NF1* and *PTPN11* post-transplant (Figure 2).

## 4. Discussion

Liver transplant as a treatment for primary and secondary liver malignancy has grown in volume, with expansion from HCC to CCA and, more recently, to CRLM [6]. However, recurrence after LT remains a concern [22]. CtDNA has emerged as a non-invasive surveillance tool in predicting and detecting recurrence after the treatment of hepatic malignancies [23]. Compared to traditionally used tumor markers (e.g., CA19-9) which are notorious for their limited sensitivity and specificity, ctDNA offers a more individualized testing modality that can be used to predict recurrence-free survival at earlier time points, leading to guided decision-making for treatment selection [24,25].

This study demonstrates proof-of-concept for ctDNA testing in patients undergoing LT for primary and secondary liver cancers. We found a higher absolute recurrence rate in patients with positive post-transplant ctDNA. In patients who experienced recurrence, ctDNA was detected in all patients with active disease. Conversely, ctDNA was not detected in the one patient who achieved remission after recurrence. When comparing pre- vs. post-transplant ctDNA, clearance of ctDNA was observed in half of the patients who underwent sequential testing. An overall reduction in the TMB was also noted after LT. Interestingly, 30% of patients with sequential testing acquired new genomic alterations in post-transplant ctDNA, which may induce caution toward recurrent malignancy and/or the introduction of confounding genomic material that influences the interpretation of the results.

Our group previously published on the use of ctDNA in the context of hepatic resection for CRLM, showing how the detection of post-operative ctDNA was associated with an increased likelihood of disease recurrence [21]. Similarly, Tie et al. (2023) [24], Liu et al. (2023) [26], and Nishioka et al. (2022) [27] showed that post-operative ctDNA positivity predicts a reduced recurrence-free (RFS) and overall survival (OS) in patients undergoing hepatectomy for CRLM. The results of the GALAXY study further demonstrate the association of post-operative ctDNA with an increased recurrence risk and the ability to identify patients who derived benefits from adjuvant chemotherapy in patients with stage II or III CRC [28]. In patients with resected CCA, the preliminary results from Yoo at al. (2023) similarly show positive ctDNA status is predictive of a poor RFS [29]. In HCC, Wang et al. (2020) showed a reduced RFS with post-operative ctDNA assessed according to a panel of four hotspot genomic mutations in *TP53* (G747T), *CTNNB1* (A121G, C133T), and *TERT* (c.-124C>T) [30]. In the setting of liver transplant for unresectable primary liver cancer, larger scale studies by Huang et al. (2023) [23] and Jiang et al. (2022) [31] again display higher recurrence rates in patients with positive post-transplant ctDNA and decreased disease-free survival. 

The widely known limitations of tumor serum biomarkers are additionally observed in our study. Of the six patients in our study who experienced recurrence, three (#6, 7, 20) had normal serum levels of traditionally used biomarkers at time of recurrence. However, ctDNA was detected post-transplant in two of these patients (#6, 7), demonstrating a potential set of patients in whom the recurrence of HCC following LT may be predicted or detected with ctDNA. To this end, expanding the enrollment of patients undergoing post-transplant ctDNA testing and conducting serial testing at earlier time points following LT may help elucidate whether the detection of ctDNA correlates with or predicts recurrence. If shown to be of prognostic utility, ctDNA could be used to stratify patients based on their risk of recurrence and determine more targeted, individualized selection of adjuvant therapy.

In addition, we report the acquisition of new mutations post-transplant in several patients who underwent sequential tumor-agnostic ctDNA testing. Although the exact source of the ctDNA is unknown, the absence of viable tumors in the explant histopathology for at least two patients may lead us to postulate that these mutations may be of donor origin. Alternatively, they may represent somatic mutations in the setting of immunosuppression post-transplant or clonal evolution. To address this concern, tumor-informed genetic testing may be considered due to its ability to differentiate ctDNA from germline-derived variants, clonal hematopoiesis of indeterminate potential, and dd-cfDNA. Such tumor-informed tools have been developed and are actively being explored in clinical studies and trials [32]. However, these methods do have limitations in patients who have received extensive pre-LT locoregional and systemic therapy, as adequate viable tumor is necessary for tissue-informed testing. Given the uncertain origin of the novel post-LT genomic alterations, making ctDNA-based treatment decisions may be challenging in this subset. At a minimum, pre- and post-LT testing should be pursued when using tissue-agnostic testing in order to obtain a pre-transplant comparison. With expanding evidence supporting the use of ctDNA testing in liver cancers [24,25,26,27,28,29,30,31], the optimization of protocols effective at addressing the concerns regarding donor-derived alterations is warranted in future studies.

In addition to assessing for the presence of ctDNA, liquid biopsy can identify specific genes that predict patient outcomes based on cancer. For example, in HCC, *CTNNB1* and *TERT* have been shown to be two of the most commonly mutated genes and were present frequently in our cohort [33]. The presence of these two mutations, along with a mutation in *TP53*, in post-operative ctDNA has been associated with a decreased recurrence-free survival [30]. In CCA, the mutations are thought to be more heterogeneous, though mutations in *KRAS*, *IDH1/2*, *FGFR*, *ERBB2*, and *BRAF* have been noted to be more frequently mutated [34]. In colorectal cancer, mutations in *APC* and *TP53* are known to drive the transition from adenoma to adenocarcinoma [35,36,37,38]. In patient #21, the presence of these mutations post-transplant, although at lower variant allele frequencies, was detected prior to diagnosis of recurrence (Table 8). While our study was not aimed at addressing the prognostic or therapeutic implications of specific genes, the correlation between our findings in solid organ transplant patients and the published findings in the non-transplant population is encouraging for the application of liquid biopsy to this new set of patients. Tissue-agnostic ctDNA testing could theoretically provide such analysis before transplant, allowing for pre-transplant prognostication. One example of potential utility is the detection of mutations that are contraindications to transplant, such as *BRAF V600E*, which represents a contraindication to LT for CRLM in our center. As detection of such a mutation pre-LT may preclude transplant due to high risk of recurrence, the use of ctDNA in the transplant population warrants further investigation for optimization of protocols and interpretation. 

The limitations of this study include a small sample size, which is insufficient for determining causal relationships between ctDNA clearance and liver transplant. Furthermore, a low number of patients had sequential testing, which interferes with the evaluation of donor-derived cell-free DNA. Inconsistency in the ctDNA sampling and timing may have arisen due to challenges in clinical practice and logistics. To address these issues, a large-scale multi-institutional study is being conducted to increase the patient volume, and new institutional protocols have been implemented to ensure adequate sampling. Furthermore, the impact of neoadjuvant and adjuvant chemo, immune, and radiation therapy on the ctDNA results is still unknown. Lastly, the correlation of ctDNA with tissue-based mutational profiles was not assessed in the present study, although concurrent tissue testing is now ongoing.

## 5. Conclusions

Circulating tumor DNA can help us to identify recurrence after liver transplant for hepatic malignancy. Transplantation was also associated with clearance of the ctDNA burden in half of the patients with sequential testing. We report a subset of patients with non-viable tumors and novel post-transplant genomic profiles, raising concern about donor-derived sources; improved approaches are necessary to address the potential of such findings confounding treatment decisions. Larger-scale studies and serial monitoring should be conducted to confirm the utility of ctDNA as a surveillance tool for MRD post-transplant and optimize the timing of the screening protocols.

## Figures and Tables

**Table 1:** Summary of demographic and pre-transplant variables and post-transplant outcomes.

	ALL N = 21	HCC N = 9	HCC/CCA N = 1	CCA N = 3	CRLM N = 8
Male Sex, N (%)	16 (76%)	8 (89%)	0	2 (67%)	6 (75%)
Race, N (%) White Black Other/Unknown	18 (86%) 2 (10%) 1 (5%)	8 (89%) 0 1 (11%)	1 (100%) 0 0	2 (50%) 1 (25%) 0	7 (88%) 1 (13%) 0
Age at Transplant Surgery, Median (IQR)	55 (50–68)	70 (46–73)	60	51 (25–55)	54 (49–60)
Cirrhosis, N (%) Non-Malignancy Cirrhosis Factors A1AT ETOH HBV HCV NASH PSC Biliary Atresia Chemotherapy-Induced PBC	19 (90%) 1 (5%) 2 (10%) 3 (14%) 1 (5%) 6 (29%) 2 (10%) 2 (10%) 1 (5%) 1 (5%)	9 (100%) 0 1 (11%) 2 (22%) 1 (11%) 5 (56%) 0 2 (22%) 0 0	1 (100%) 1 (100%) 0 1 (100%) 0 0 0 0 0 0	3 (75%) 0 1 (25%) 0 0 0 1 (25%) 0 0 0	6 (86%) 0 0 0 0 1 (14%) 1 (14%) 0 1 (14%) 1 (14%)
MELD Score, Median (IQR)	15 (11–24)	22 (14–25)	24	12 (10–29)	11 (7–19)
Pre-Treatment Tumor Marker Level, Mean (SD) AFP (ng/mL) CA19-9 (U/mL) CEA (ng/mL)	8 (6, 14) 23 (12, 168) 31 (1, 64)	8 (6, 13)	39 216	22 (8, 24)	31 (1, 64)
Pre-Transplant Number of Lesions, N (%) 1 2–3 Innumerable	11 (52%) 6 (29%) 2 (10%)	7 (78%) 1 (11%) 0	0 1 (100%) 0	4 (100%) 0 0	0 4 (50%) 2 (25%)
Pre-Transplant Size of Biggest Lesion (cm), Median (IQR)	4 (2–6)	1 (1–4)	3	1	6.7 (3–8)
Pre-Transplant Treatment, N (%) Systemic Chemotherapy Radiotherapy SBRT Prior Surgery Ablation Chemoembolization Radioembolization Immunotherapy	18 (86%) 10 (48%) 6 (29%%) 4 (19%) 3 (14%) 4 (19%) 6 (29%) 5 (24%) 1 (5%)	7 (78%) 0 0 0 0 1 (11%) 2 (22%) 4 (44%) 0	0 0 0 0 0 0 0 0 0	3 (100%) 2 (67%) 2 (67%) 2 (67%) 0 0 0 0 0	8 (100%) 8 (100%) 4 (50%) 2 (25%) 3 (38%) 3 (38%) 4 (50%) 1 (13%) 1 (13%)
Post-Transplant Tumor Marker Level, Median (IQR) AFP (ng/mL) CA19-9 (U/mL) CEA (ng/mL)	3 (3–7) 13 (6–20) 2 (1–2)	3 (3–6.8)	4.8	13 (6–20)	1.7 (1–2)
Recurrence, N (%)	6 (29%)	3 (33%)	0	1 (25%)	2 (25%)
Patient Status, N (%) Alive Dead	17 (81%) 4 (19%)	6 (67%) 3 (33%)	1 (100%) 0	2 (67%) 1 (33%)	8 (100%) 0
Cancer-Related Deaths, N (%)	2 (10%)	2 (22%)	0	0	0
Recurrence Survival (Days), Median (IQR) Overall Survival (Days), Median (IQR)	13 (7–28) 14 (8–39)	12 (5–31) 14 (6–34)	16.7 16.7	8 (6–15) 8 (6–32)	14 (10–40) 25 (10–60)

Key: A1AT = alpha-1-anti-trypsin deficiency, ETOH = ethanol, HBV = hepatitis B virus, HCV = hepatitis C virus, NASH = non-alcoholic steatohepatitis, PSC = primary sclerosing cholangitis, PBC = primary biliary cholangitis, AFP = alpha-fetoprotein, CA19-9 = cancer antigen 19-9, CEA = carcinoembryonic antigen, SBRT = stereotactic body radiation therapy.

**Table 2:** Transplant variables.

Pt	Age	Sex	Cancer Type	Cirrhosis Factors	MELD at Tx	Tx Type	Liver Transplant Technique	Aberrant Liver Vasculature	Type of Arterial Anastomosis	Type of Venous Anastomosis	Biliary Anastomosis	Real Warm Ischemia Time (min)	LT Duration (min)	RBCs, FFP (Units)	Reperfusion Order	Post-Reperfusion Syndrome
**1**	39	M	HCC	Biliary atresia, PBC	12	LDLT	Piggyback	-	Standard	Interposition	HJ	38	776	0, 0	Vein first	No
**2**	70	M	HCC	NASH	22	DCD	Piggyback	-	Standard	End-to-end	Duct-to-duct	43	541	6, 3	Vein first	No
**3**	52	M	HCC	HCV	23	DCD	Conventional	Replaced RHA	Standard	End-to-end	Duct-to-duct	-	-	-	Vein first	Yes
**4**	75	M	HCC	NASH	25	DBD	Piggyback	-	Standard	End-to-end	Duct-to-duct	46	505	2, 0	Vein first	No
**5**	66	M	HCC	NASH, ETOH, HBV	9	LDLT	Piggyback	Accessory LHA	Standard	End-to-end	Duct-to-duct	39	720	0, 0	Vein first	No
**6**	70	M	HCC	HBV	25	DBD	Piggyback	Accessory RHA	Standard	End-to-end	Duct-to-duct	46	430	0, 0	Vein first	No
**7**	72	M	HCC	NASH	11	LDLT	Piggyback	-	Standard	End-to-end	Duct-to-duct	42	557	7, 5	Vein first	No
**8**	73	M	HCC	NASH	12	DCD	Piggyback	-	Standard	End-to-end	Duct-to-duct	58	410	4, 1	Both	No
**9**	32	F	HCC	Biliary atresia	40	Split, DBD	Conventional	-	Standard	End-to-end	HJ	45	657	18, 13	Vein first	No
**10**	60	F	HCC/CCA	HBV, A1AT	24	DCD	Piggyback	-	Standard	End-to-end	Duct-to-duct	48	385	8, 1	Both	Yes
**11**	25	M	CCA	PSC	12	DCD	Conventional	-	Standard	End-to-end	HJ	42	490	3, 0	Vein first	Yes
**12**	51	F	CCA	ETOH	29	DBD	Piggyback	Replaced LHA	Standard	Conduit	HJ	27	683	17, 11	Vein first	Yes
**13**	55	M	CCA	-	10	DBD	Conventional	Replaced RHA	Infra-renal	Conduit	HJ	40	452	1, 0	Vein first	No
**14**	50	M	CRLM	-	6	LDLT	Piggyback	-	Standard	End-to-end	Duct-to-duct	39	584	0, 0	Vein first	No
**15**	53	M	CRLM	-	6	DBD	Conventional	-	Standard	End-to-end	HJ	49	427	0, 0	Vein first	Yes
**16**	61	M	CRLM	-	13	LDLT	Conventional	-	Standard	End-to-end	Duct-to-duct	32	869	4, 0	Vein first	No
**17**	64	M	CRLM	-	11	LDLT	Piggyback	-	Standard	End-to-end	Duct-to-duct	43	594	7, 8	Vein first	No
**18**	54	M	CRLM	-	14	LDLT	Piggyback	-	Standard	Interposition	Duct-to-duct	67	992	5, 0	Vein first	No
**19**	49	F	CRLM	PBC	23	LDLT	Piggyback	-	Standard	End-to-end	Duct-to-duct	27	685	2, 0	Vein first	No
**20**	49	M	CRLM	-	21	DBD	Piggyback	-	Standard	End-to-end	HJ	39	708	20, 12	Vein first	No
**21**	56	F	CRLM	NASH	8	LDLT	Piggyback	-	Standard	Interposition	Duct-to-duct	45	700	4, 0	Vein first	Yes

Key: PBC = primary biliary cholangitis, NASH = non-alcoholic steatohepatitis, HCV = hepatitis C virus, ETOH = ethanol, HBV = hepatitis B virus, A1AT = alpha-1 anti-trypsin deficiency, PSC = primary sclerosing cholangitis, LDLT = living donor liver transplant, DCD = donation after cardiac death, DBD = donation after brain death, RHA = right hepatic artery, LHA = left hepatic artery, HJ = hepaticojejunostomy.

**Table 3:** Post-transplant variables.

Patient	Induction IS	Initial IS	IS 12 month	Biliary Complications	Biliary Intervention	Arterial Complications, Intervention	Acute Rejection Grade	Treatment of Acute Rejection	Chronic Rejection
**1**	Basiliximab	GC + Tacrolimus	-	Leak	PTHC	-	-	-	-
**2**	-	GC + MMF + Tacrolimus	-	-	-	-	-	-	-
**3**	Basiliximab	GC + Tacrolimus	Tacrolimus + Sirolimus	-	-	-	-	-	-
**4**	Basiliximab	GC + MMF + Tacrolimus	Cyclosporine + Everolimus	-	-	-	-	-	-
**5**	Basiliximab	GC + MMF + Tacrolimus	Tacrolimus + MMF	Leak	ERCP	-	Mild	IV steroids	-
**6**	Basiliximab	GC + MMF + Tacrolimus	Tacrolimus + MMF + Sirolimus	-	-	-	-	-	-
**7**	Basiliximab	GC + MMF + Tacrolimus	-	-	-	-	-	-	-
**8**	-	GC + MMF + Tacrolimus	Tacrolimus + Everolimus	Stricture	ERCP	-	-	-	-
**9**	-	GC + MMF + Tacrolimus	-	-	-	-	-	-	-
**10**	Basiliximab	GC + MMF + Tacrolimus	Tacrolimus + Everolimus	-	-	-	-	-	-
**11**	-	GC + MMF + Tacrolimus	-	Leak, ischemic cholangiopathy	HJ reconstruction, PTHC, re-transplant	HA stenosis and pseudoaneurysm, stent placement	-	-	-
**12**	Basiliximab	GC + MMF + Tacrolimus	Tacrolimus + GC + MMF	-	-	-	-	-	-
**13**	-	GC + MMF + Tacrolimus	-	-	-	-	-	-	-
**14**	Basiliximab	GC + MMF + Tacrolimus	-	-	-	-	-	-	-
**15**	-	GC + MMF + Tacrolimus	Tacrolimus + Everolimus	Leak	Re-operation	-	-	-	-
**16**	Basiliximab	GC + MMF + Tacrolimus	-	Stricture	ERCP	-	Mild	IV steroids	-
**17**	Basiliximab	GC + MMF + Tacrolimus	Tacrolimus + Everolimus	Leak	ERCP + PTC	-	-	-	-
**18**	Basiliximab	GC + MMF + Tacrolimus	Tacrolimus + Everolimus	Stricture	ERCP	-	-	-	-
**19**	-	GC + MMF + Tacrolimus	-	-	-	-	-	-	-
**20**	-	GC + MMF + Tacrolimus	-	-	-	-	-	-	-
**21**	-	GC + MMF + Tacrolimus	-	-	-	-	-	-	-

**Table 4:** Tumor marker correlation with ctDNA testing at times prior to and following transplant, along with time of recurrence.

Pt	Cancer Type	Date Liver Cancer dx	Dx Date Tumor Marker Level	Dx Tumor Marker Level	Date Pre-Transplant Marker	Pre-Transplant Tumor Marker	Date of Pre-Transplant ctDNA	Pre- ctDNA Results	Date of Transplant	Date Post-Transplant Marker	Post-Transplant Tumor Marker	Date of Post-Transplant ctDNA	Post-Transplant ctDNA Results	ctDNA Timing	Date of Recurrence	Date of Tumor Marker Level with Recurrence	Recurrence Tumor Marker Level
**1**	HCC	7/2022	8/2/2023	AFP: 15	8/15/23	AFP: 24.1	8/15/23	+ (CDx)	8/21/23	10/26/23	AFP: <3.0						
**2**	HCC	2/16/2023	4/17/2023	AFP: <3	4/17/2023	AFP: <3	4/19/2023	+ (CDx)	6/9/2023	12/5/23	AFP: <3.0	6/20/23	+	Both			
**3**	HCC	8/10/2010	8/18/2010	AFP: 8.7	9/24/2010	AFP: 4.6			10/12/2010	10/21/2010	AFP:7.1	12/19/2019	+	Post-	12/16/2019	12/17/2019	AFP: 4398.6
**4**	HCC	12/18/2020	12/18/2020	AFP: 6.2	3/7/2022	AFP: 9.3			4/9/2022	6/21/2022	AFP: <3.0	4/11/2023	+	Post-			
**5**	HCC	7/11/2022	1/27/2022	AFP: 11	1/27/2022	AFP: 11			7/11/2022	7/28/2022	AFP: <3.0	8/10/2022	+	Post-			
**6**	HCC	2/18/2019	2/18/2019	AFP: 7.6	2/3/2020	AFP: 4.9			5/1/2020	10/23/2020	AFP: <3	11/12/2021	+	Post-	10/23/2020	6/2/21	AFP: <3.0
**7**	HCC	3/15/2022	3/15/2022	AFP: 7.1	09/08/2022	AFP: 9.5			9/18/2022	10/10/2022	AFP: 10.5	11/14/2022	+	Post-	10/4/2023	10/4/23	AFP: <3.0
**8**	HCC	11/8/2019	8/2/2019	AFP: 5.6	2/20/2020	AFP: 6.3	12/18/2019	-	4/12/2020	12/16/2020	AFP: <3			Pre-			
**9**	HCC	7/19/2022	7/19/2022	AFP: 14	12/8/2022	AFP: 8.1	8/15/2022	+	12/30/2022	11/18/2022	AFP: 6	9/1/2023	-	Both			
**10**	HCC/CCA	5/4/2021	5/4/2021; 5/20/2021	AFP: 38.8, CA19-9: 216	7/5/2022	AFP: 36.6, CA 19-9: 834	6/6/2022	+	7/7/2022	7/22/2022	AFP: 4.8			Pre-			
**11**	CCA	7/14/2021	6/9/2021	CA19-9: 8	1/12/2023	CA 19-9: 46	7/20/2022, 9/2/22	+, +	2/7/2023, 07/13/23			9/28/2023	-	Both			
**12**	CCA	7/3/2020	6/19/2020	CA19-9: 22	1/10/2023	CA 19-9: 15			3/1/2023	7/31/2023	CA19-9: 6.3	6/14/2022	+	Post-	11/3/2021	10/25/21; 5/25/21	CA 19-9: 146; AFP: <3
**13**	CCA	11/25/2022	1/21/2021	CA19-9: 24	8/4/2020	CA 19-9: 45	12/13/2022	-	8/6/2020	12/1/2020	CA19-9: 20	8/1/2023	-	Both			
**14**	CRLM	6/2017	12/10/2018	CEA: 2.4	9/21/22	CEA: 1	10/27/22, 9/25/23	-, -	10/11/23	12/11/23	CEA: 1.6	11/1/23	+	Both			
**15**	CRLM	2/20/2020	3/3/2020	CEA: 6854	8/9/2022	CEA: 4.9	5/19/2022	+	9/14/2022	1/10/2023	CEA: 1.8	11/15/2022	- (GR)	Both			
**16**	CRLM	10/5/2017	9/15/2017	CEA: 60.1	1/6/2020	CEA: 10.4	11/11/2019	+	1/12/2020	2/6/2020	CEA: 1.2	1/12/2022, 7/15/22, 1/16/23	-, -, - (GR)	Both			
**17**	CRLM	2019	8/26/2021	CEA: 1	10/28/2022	CEA: 2.7	11/1/2022	+	11/1/2022	12/15/2022	CEA: 0.9	12/8/2022, 6/7/23	+, +	Both			
**18**	CRLM	11/12/2011	8/23/2011	CEA: 1.6	9/10/2020	CEA: 1.6	6/25/2019	+	9/13/2020	1/9/2023	CEA: 1.7	11/8/2021, 5/5/22	- (GR)	Both			
**19**	CRLM	4/1/2016	4/14/2016	CEA: 64.4	11/27/2017	CEA: 3.7			4/22/2018	5/24/2018	CEA: 3.8	1/23/2020, 4/19/22	+, +	Post-			
**20**	CRLM	6/16/2012	N/A	N/A	5/27/2018	CEA: 2.9			5/27/2018	8/30/2018	CEA: 2	5/27/2022, 2/23/22, 7/28/23	-, -, - (GR)	Post-	9/19/2019	9/19/2019	CEA: 1.8
**21**	CRLM	11/9/2020	11/2/2020	CEA: 30.7	8/9/2022		8/6/2022, 11/10/22	+, -	2/6/2023	7/3/23	CEA: 16.5	10/31/23	+	Both	9/25/23	7/17/23; 9/25/23	CEA: 17.2; CEA: 17.9

Key: CDx = Guardant CDx; GR = Guardant Reveal. All other ctDNA results are from Guardant360. AFP units = ng/mL, CA19-9 units = U/mL, CEA units = ng/mL. “+” and “-” symbols correspond to presence or absence of ctDNA respectively.

**Table 5:** Oncologic variables including treatment before and after liver transplant as well as with recurrence.

Pt	Cancer Type	Liver Cancer dx	Pre-Transplant Treatment	Chemotherapy Details	Radiation Therapy Details	Surgery Details	Pathologic Response	Date of Transplant	Date of Recurrence	Recurrence, Number of Tumors, Sites	Largest Tumor Size (cm)	Treatment of Recurrence	Recurrence Treatment Details	Death, Cause
**1**	HCC	07/2022	TARE		9/2022		PR	8/15/23						
**2**	HCC	2/16/2023	-					6/9/2023		No				
**3**	HCC	8/10/2010	Microwave ablation					10/12/2010	12/16/2019	Intrahepatic; multifocal	11.5	Chemotherapy	02/2/2020–11/6/2020: levatinib; switched to cobozanrinib after progression until 11/6/2020	12/17/2020; HCC
**4**	HCC	12/18/2020	TACE, TARE	03/2/12, 5/11/21, 8/18/21	1/14/22		PR	4/9/2022		No				
**5**	HCC	7/11/2022	-					7/11/2022		No				
**6**	HCC	2/18/2019	TARE		09/19/2019, 11/19/2019		SD	5/1/2020	10/23/2020	Extrahepatic; multifocal—lung, adrenal fossa, retrocaval lymph nodes	1.3	Chemotherapy, radiation	9/7/21: radiation; 12/3/21–2/3/22: levatinib	5/13/2022: HCC
**7**	HCC	3/15/2022	TARE		5/18/22		SD	9/18/2022	10/4/2023	Extrahepatic; multifocal—lung	1.5	Chemotherapy	11/22/23: levatinib	
**8**	HCC	11/8/2019	TACE	12/2/2019			PR	4/12/2020		No				10/8/2023: metastatic melanoma
**9**	HCC	7/19/2022	SBRT	09/26/22–10/10/22: 4 treatments			PR	12/30/2022		No				
**10**	HCC/CCA	5/4/2021	-					7/7/2022		No				
**11**	CCA	7/14/2021	Chemoradiation	08/29/22–09/16/22: capecitabine	08/29/22–09/16/22		SD	2/7/2023, 07/13/23		No				
**12**	CCA	7/3/2020	SBRT	09/26/2019–09/27/2019			CR	8/6/2020	11/3/2021	Extrahepatic; multifocal—liver, bone		Chemotherapy, radiation	9/6/22–9/21/22: radiation; 12/1/21–7/1/22: gemcitabine/oxaliplatin; 7/26/22–8/1/22: FOLFIRI; 10/1/22–12/1/22: gemcitabine/abraxane x 3 with PR	3/15/23: cardiovascular event during dialysis; CCA
**13**	CCA	11/25/2022	Chemoradiation, SBRT	1/10/23–2/3/23: capecitabine	1/10/23–2/3/23		CR	3/1/2023, adjuvant capecitabine x 4 cycles (6/5/23)		No				
**14**	CRLM	2015	Chemotherapy, surgery, microwave ablation	7/11/17–9/20/17, 8/2019–5/8/2018: FOLFOX/cetuximab; 8/19–2/20: capecitabine, 8 cycles; 4/19/21–9/21: capecitabine	5/18/22: microwave ablation; 2/2/23: SBRT 30 Gy in 1 fraction	12/12/2017: open wedge resection (segments 4–8); 1/18/19: segment 4b lesion resection; 7/2/19: segment 8 lesion resection; 2/23/21: segments 7/8 liver resection	CR	10/11/23		No				
**15**	CRLM	2/20/2020	Chemotherapy, immunotherapy, radiation therapy	3/20–8/18/20: CAPOX, bevacizumab; 10/2020–early 2021: 5FU, bevacizumab; 07–08/21: 5FU only; 10/21–01/22: 5FU, bevacizumab	01–06/2021		CR	9/14/2022		No				
**16**	CRLM	10/5/2017	Chemotherapy, TARE	10/2017–02/2018: FOLFOX, Avastin x 9 cycles; 02/18–12/11/19: FOLFIRI/panitumumab	4 rounds		PR	1/12/2020		No				
**17**	CRLM	2019	Chemotherapy, radiation therapy, SBRT	09–11/11/2020: FOLFOX, Avastin x 12 cycles; 12/2020–05/2021: Avastin	SBRT: 9/20/2020		CR	11/1/2022		No				
**18**	CRLM	11/12/2011	Chemotherapy, radiation therapy, surgery, TACE, RFA	10/18/2011–04/2012: Xeloda, FOLFIRI x 3 cycles; 07/2017: FOLFIRI, Erbitux; 02/25/15–03/2015: HAI pump infusion therapy		Hepatic resection 02/25/2015 and 09/2016	PR	9/13/2020		No				
**19**	CRLM	4/1/2016	Surgery, TACE, chemotherapy	1/17/2015: HAI FUDR; 8/26/2016: FOLFIRI w/ panitumumab x 6 cycles, FOLFOX Avastin x 3 cycles		Wedge resection segments 2 and 3, caudate lobe removal, R hepatectomy	CR	4/22/2018		No				
**20**	CRLM	6/16/2012	Chemotherapy, ablation, TACE, radiotherapy	08–10/2013: FOLFIRI; 12/2013: hepatic resection, HAI pump; until 10/2014: FUDR; 01–04/2014: 5FU; 05–01/2016: irinotecan, cetuximab; 02/2016–11/2017: 5FU cetuximab, 3/7/2018: FOLFOX x 13 cycles	12/2017: proton beam radiotherapy	07/2013: Ablation	*	5/27/2018	9/19/2019	Extrahepatic; unifocal, right upper lobe of lung	0.9	Chemotherapy, surgery	Right upper lobe metastectomy; 12/16/2019–7/27/2020: FOLFIRI, bevacizumab with complete response	
**21**	CRLM	11/9/2020	Chemotherapy, TACE	5/2021: FOLFOX x 7 cycles; 6/28/22–11/7/22: irinotecan; 9/28/22–1/4/23: panitumumab; 3/2/22: infusional 5FU			PR	2/6/23	9/25/23	Intrahepatic and extrahepatic—lung nodule		Chemotherapy, plan for surgery	10/17/23: irinotecan, panitumumab	

Key: SBRT = stereotactic radiation body therapy, TACE = trans-arterial chemoembolization, TARE = trans-arterial radioembolization, RFA = radiofrequency ablation, PR = partial response, CR = complete response, SD = stable disease. * = Information at OSH.

**Table 6:** Pre- vs. post-transplant mutational profiles of patients who underwent sequential ctDNA testing by cancer type.

Patient #	Cancer Type	Time From Pre-op Testing to Surgery (Days)	Pre-op Somatic Alterations Detected	Pre-Transplant ctDNA	Time from Surgery to Post-op Testing (Days)	Post-op Somatic Alterations Detected	Post-Transplant ctDNA
2	HCC	51	Yes	CTNNB1 L31V 0.20%	11	Yes	CTNNB1 D32V N/A
9	HCC	137	Yes	*TERT* Promoter SNV 0.80% *FGFR2* K509E 2.00%	245	No	Not Identified
11	HCC	148	Yes	Not Identified	233	No	Not Identified
13	CCA	78	No	Not Identified	153	No	Not Identified
14	CRLM	16	No	Not Identified	21	Yes	*ROS1* L1899F 0.2%
15	CRLM	26	Yes	*MTOR* Q1715 0.40%	62	No	Not Identified
16	CRLM	62	Yes	*APC* E1064 * 0.50% *TP53* R248Q 0.10% *SMAD4* A418fs 0.06% *MAP2K1* K84R 0.20%	731	No	Not Identified
17	CRLM	0	Yes	*NF1* A706V 0.10% *MLH1* I191 0.20%	37	Yes	*FGFR3* T317A 1.80% *PALB2* N241D 1.60% *BRCA2* C1290Y 1.50% *ROS1* T632N 1.20% *MET* V378I 0.10%
18	CRLM	293	Yes	*ROS1* A2106T 0.20% *BRCA1* K22E 0.10%	421	No	Not Identified
21	CRLM	184	Yes	*APC* S1415fs 1% *TP53* S149fs 1.3%	266	Yes	*APC* S1415fs 0.2% *TP53* S149fs 0.2%

Note: Percentages shown represent %cfDNA (cell-free DNA). N/A = not available. Asterisk (*) indicates unknown substitution.

**Table 7:** Tumor details from diagnostic radiologic imaging and explant pathology.

Pt	Cancer Type	DxNumber of Tumors	Dx-Largest Tumor Size (cm)	Pathologic Tumor Numbers	Pathologic Largest Tumor Size (Viable) (cm)	% Viable Tumor Explanted Liver	Pathologic Vascular Invasion	Pathologic Perineural Invasion	Pathologic Liver Capsule Involvement	Histologic Grade of Differentiation	MSI	Pathologic TNM Staging from Transplant
**1**	HCC	1	3.6	3	0.8	20%	Small vessel	Absent	Absent	G2		T2
**2**	HCC	1	6.6	1	2.5	100%	Absent	Absent	Absent	G2		T1b
**3**	HCC	1	4	1	3	0%	Absent	Absent	Absent	G2		T1bN0
**4**	HCC	3	2.8	1	2.3	100%	Absent	Absent	Absent	G2		T2
**5**	HCC	5	1.7	5	1.7	100%	Small vessel	Absent	Absent	G2–3		T2
**6**	HCC	1	4.9	4	2.7	5%	Small vessel	Absent	Absent	G2		T2N0
**7**	HCC	1	4.2	Multiple	4.3	50%	Small and large vessel	Absent	Abuts	G2		T4
**8**	HCC	1	2.6	1	0.8	50%	Absent	Absent	Absent	G2		T1a
**9**	HCC	1	8	1	2.3	20%	Absent	Absent	Absent	G2		T1b
**10**	HCC/CCA	3	2.3	3 (2-HCC, 1-CCA)	2-HCC, 10-CCA	0%, 5%, 95%	Present	Present	Posterior capsule	G2–3		T2
**11**	CCA	1	1	1	0.1	100%	Absent	Absent	Absent	G1		T2aN0
**12**	CCA	1	1	0	0	N/A	N/A	N/A	N/A	N/A		
**13**	CCA	1	1	1 (residual)	No gross lesion visible					G2		T1N0
**14**	CRLM	Numerous	7.6	0	0	N/A	N/A	N/A	N/A	N/A	Stable	T0N1aM1
**15**	CRLM	Numerous	7.7	21	4.1	20%	Absent	Absent	Absent		Stable	T3N1M1a
**16**	CRLM	3	5.8	3	4	100%, 0%	Absent	Absent	Absent		Stable	T3N1aM1
**17**	CRLM	*	*	1	8.5	0%	Absent	Absent	Absent		Stable	T3N1aM1
**18**	CRLM	3	*	1	4	0%	Absent	Absent	Absent		Stable	
**19**	CRLM	2	*	0							Stable	T3N1aM1
**20**	CRLM	*	*	4	1.7	100%	Absent	Absent	Absent	G2	Unknown	
**21**	CRLM	2	1.4	6	3.3	100%	Absent	Absent	Absent	G2	Stable	T3N0M1

Key: * = imaging performed at OSH, Dx = diagnostic, N/A = not applicable.

**Table 8:** ctDNA profiles for patients who experienced recurrence.

Patient Number	Cancer Type	Date Pre-Transplant ctDNA Collected	Pre-op Somatic Alterations Detected	Pre-Transplant ctDNA	Date Post-Transplant ctDNA Collected	Post-op Somatic Alterations Detected	Post-Transplant ctDNA	Date of Recurrence
3	HCC				12/19/2019	Yes	*CTNNB1* T41A 3.70% *TERT* Promoter 2.00%	12/16/2019
6	HCC				11/12/21	Yes	*ARID1A* S696fs 0.70% *CTNNB1* S33A 16.50% *TERT* promoter 13.30%	10/23/2020
7	HCC				11/14/2022	Yes	*TP53* R248Q 0.10% *FGFR1* V247V 6.00%	10/4/2023
12	CCA	12/3/22	No	Not identified	8/1/23	No	Not identified	11/3/2021
20	CRLM				7/28/23	No	Not Identified	9/19/2019
21	CRLM	8/6/22	Yes	*TP53* S149fs 1.30% *APC* S1415fs 1.00% *AR* R780W 0.50%	10/31/23	Yes	*APC* S1415fs 0.2% *TP53* S149fs 0.2%	9/25/23

Note: Percentages shown represent %cfDNA (cell-free DNA).

## Data Availability

The data presented in the study are available in the paper.
